# Microfluidic ion stripper for removal of trifluoroacetic acid from mobile phases used in HILIC-MS of intact proteins

**DOI:** 10.1007/s00216-021-03414-4

**Published:** 2021-05-28

**Authors:** Sam Wouters, Sebastiaan Eeltink, Rob Haselberg, Govert W. Somsen, Andrea F. G. Gargano

**Affiliations:** 1 Department of Chemical Engineering, Vrije Universiteit Brussel (VUB), 1050 Brussels, Belgium; 2 Center for Analytical Sciences Amsterdam, Science Park 904, 1098 XH Amsterdam, The Netherlands; 3 Division of BioAnalytical Chemistry, Amsterdam Institute of Molecular and Life Sciences, Vrije Universiteit Amsterdam, de Boelelaan 1085, 1081 HV Amsterdam, The Netherlands

**Keywords:** Microfluidic chips, LC-MS, MS ion suppression, LC ion-pairing, TFA, Intact protein analysis

## Abstract

Trifluoroacetic acid (TFA) is commonly used as mobile phase additive to improve retention and peak shape characteristics in hydrophilic interaction liquid chromatography (HILIC) of intact proteins. However, when using electrospray ionization-mass spectrometry (ESI-MS) detection, TFA may cause ionization suppression and adduct formation, leading to reduced analyte sensitivity. To address this, we describe a membrane-based microfluidic chip with multiple parallel channels for the selective post-column removal of TFA anions from HILIC. An anion-exchange membrane was used to physically separate the column effluent from a stripper flow solution comprising acetonitrile, formic acid, and propionic acid. The exchange of ions allowed the post-column removal of TFA used during HILIC separation of model proteins. The multichannel design of the device allows the use of flow rates of 0.2 mL/min without the need for a flow splitter, using mobile phases containing 0.1% TFA (13 mM). Separation selectivity and efficiency were maintained (with minor band broadening effects) while increasing the signal intensity and peak areas by improving ionization and reducing TFA adduct formation.

**Supplementary Information:**

The online version contains supplementary material available at 10.1007/s00216-021-03414-4.

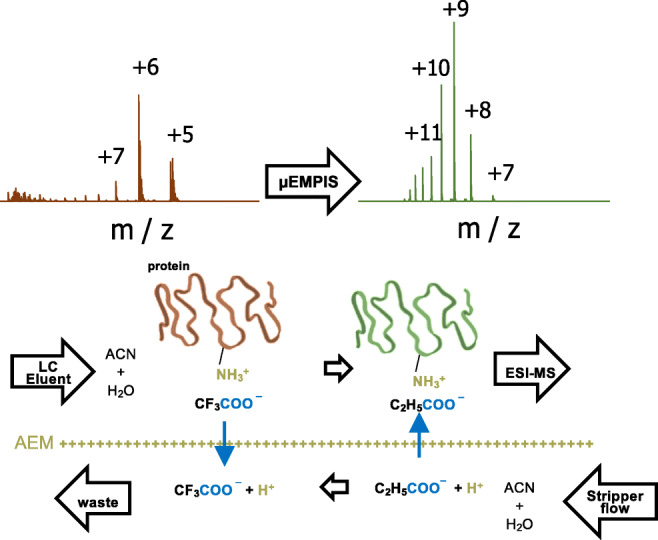

## Introduction

A clear advantage of workflows that study intact proteins by LC-MS compared to bottom-up proteomics is their capacity of maintaining information about the distribution of proteoforms [1]. This includes, for example, sequence variants and post-translational modifications (PTMs), such as glycosylation and phosphorylation [2]. Proteoforms of, e.g., industrial enzymes or therapeutic proteins may have a different activity. Therefore, increasing attention is currently devoted to developing LC-MS methods that allow characterization of proteins using so-called top-down proteomics analytical workflows, i.e., leaving the protein intact during analysis [3].

Reversed-phase liquid chromatography (RPLC) using acidic water-acetonitrile (ACN) gradients is commonly used for the denaturing LC-MS analysis of intact proteins. In addition, hydrophilic interaction liquid chromatography (HILIC)-MS of intact proteins has been gaining attention due to its unique selectivity, being complementary to RPLC and thereby providing possibilities for protein glycoform analysis. [4–8] Both in RPLC and HILIC, the use of ion-pairing agents is essential in order to circumvent adverse ionic interactions of proteins with the stationary phase and allow separations to be based solely only on hydrophobic (RPLC) or hydrophilic interactions (HILIC). Strong ion-pairing agents like trifluoroacetic acid (TFA) can modulate retention, improve separation efficiency, and minimize peak tailing. In HILIC, they also facilitate protein solubilization and recovery [9, 10].

However, TFA often impairs ESI-MS detection to an extent that may vary between mass spectrometry type and generation of instruments. Typically, TFA in the mobile phase causes a shift in the protein ion charge state distribution to lower charge states as compared to more volatile acids like formic acid. Moreover, the protein signal may be distributed over multiple TFA adducts [11]. This is exemplified in Fig. S1 of the Supplementary Information (ESM). To address this, several post-column strategies to perform efficient LC-MS using TFA-containing mobile phases have been proposed. These approaches include dilution [12–14], electrophoretic mobility control [15], membrane-based dialysis [16, 17], and exposure of the nebulized LC effluent to gas vapors (e.g., ACN, propionic acid) during ESI [18, 19]. Most of these reports are limited to low-flow applications, use flow splitters, or require complicated setups, hindering their general use. Protein-TFA adduct formation during ESI can be counteracted by applying (in-source) collision-induced dissociation at increased energies (e.g., 50 eV or more) [20]. However, this approach does not counteract ion suppression and may lead to in-source fragmentation of the protein.

Previously, we have developed a single-channel microfluidic membrane suppressor for use in capillary ion chromatography [21, 22]. In this study, we describe the design and application of a multichannel microfluidic suppressor (μEMPIS: microfluidic ESI-enabling Mobile Phase Ion-Strip) for the selective removal of TFA ions from LC mobile phases, and exchange with propionic acid and formic acid. The performance of the μEMPIS is evaluated for HILIC separations of selected intact proteins, investigating operating conditions and their influence on the dispersion and MS gains using separation flow rates up to 0.2 mL/min.

## Experimental

### Chemicals

Deionized water (18.2 mΩ) was obtained from a Milli-Q purification system (Millipore, Bedford, USA). Acetonitrile (ACN, MS grade), isopropanol (IPA, LC-MS grade), formic acid (FA), propionic acid (PA), and trifluoroacetic acid (TFA, MS grade) were obtained from Biosolve (Valkenswaard, The Netherlands). Cytochrome *C* (Cyt *C*, equine heart, ≥95%), carbonic anhydrase (CA, bovine erythrocytes, ≥95%), lysozyme (Lys, chicken egg white, 95%), myoglobin (Myo, horse heart, >90%), ribonuclease A (RnA, bovine pancreas Type I-A, ≥60%), ribonuclease B (RnB, bovine pancreas, ≥80%), transferrin (Trans, human, ≥ 98%), and ubiquitin (Ubi, bovine erythrocytes, ≥98%) were acquired from Sigma (Zwijndrecht, The Netherlands). Protein standard solutions (1 mg/mL) were prepared in deionized water.

### Fabrication of microfluidic TFA ion stripper (μEMPIS)

**Fig. 1:**
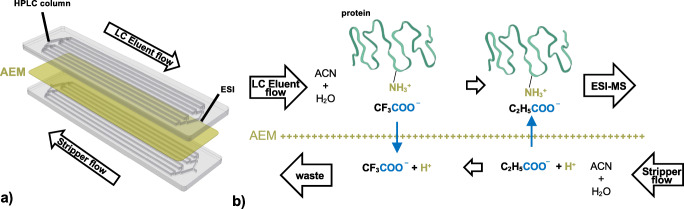
**a** Schematic representation of the post-column microfluidic device for TFA removal (μEMPIS), showing the two COC chip substrates integrating eight open channels facing each other (gray) separated by an anion-exchange membrane (yellow). The eluent from the LC separation and the stripper flow run countercurrent in the two identical COC chip substrates. The channels are flushed from a single inlet to a single outlet using a flow distributor and collector having the same configuration. The outlet of the μEMPIS is connected to ESI-MS. **b** Illustration of the ion-exchange processes taking place in correspondence of the anion-exchange membrane (AEM). The effluent flow after the HPLC separation contains TFA ions that are exchanged with PA ion carried in the stripper flow

The device was designed using AutoCAD (Autodesk, San Rafael, USA), machining jobs were programmed using PrimCAM (Primus Data, Einsiedeln, Switzerland), and parts were manufactured using an M7 Compact CNC micro mill (Datron, Mühltal, Germany). An aminated vinylbenzyl chloride anion-exchange membrane (Thermo Fisher Scientific, Sunnyvale, USA) was pre-hydrated in the stripping solution and placed between two cyclic olefin copolymer (COC) substrate plates (TOPAS grade 8007, Kunststofzentrale Leipzig, Leipzig, Germany) with dimensions 90 × 20 × 1.5 mm, featuring a microfluidic network with bifurcating flow distributor, 8 parallel channels (L = 60 mm), and flow collector (all 200 μm deep and 300 μm wide). The device volume is 32 μL. The stack was placed in a custom-made PEEK holder with aluminum backing featuring NanoPort connections (Upchurch Scientific, Oak Harbor, USA). The holder was bolted together with controlled torque. Dimensions of the μEMPIS device is 110 × 40 × 30 mm. A schematic representation of the device and its operating principle is reported in Fig. 1.

### LC separations

HILIC protein separations were performed on an Agilent HPLC 1290 Infinity II (Waldbronn, Germany), composed of a binary pump, autosampler, column thermostat, and variable wavelength detector, using an Agilent AdvanceBio Glycan Mapping column (150 × 2.1 mm, 1.8 μm particle diameter with 300 Å pore size) with a Phenomenex SecurityGuard Ultra Cartridge (Widepore C4; 2 × 4.6 mm i.d.) (Utrecht, The Netherlands).

Gradient separations were performed using solvent A: 98% ACN, 2% water, 0.1% TFA, and B: 88% water, 10% IPA, 2% ACN, 0.1% TFA. Gradient details are as follows: for intact proteins: a linear gradient from 20 to 50% B in 20 min; for the separation of RnA and RnB: 20 to 30% B in 1 min followed by a linear gradient from 30 to 37% B in 20 min. The flow rate and column temperature were 0.1 mL/min and 60 °C, respectively, unless stated differently.

**Fig. 2:**
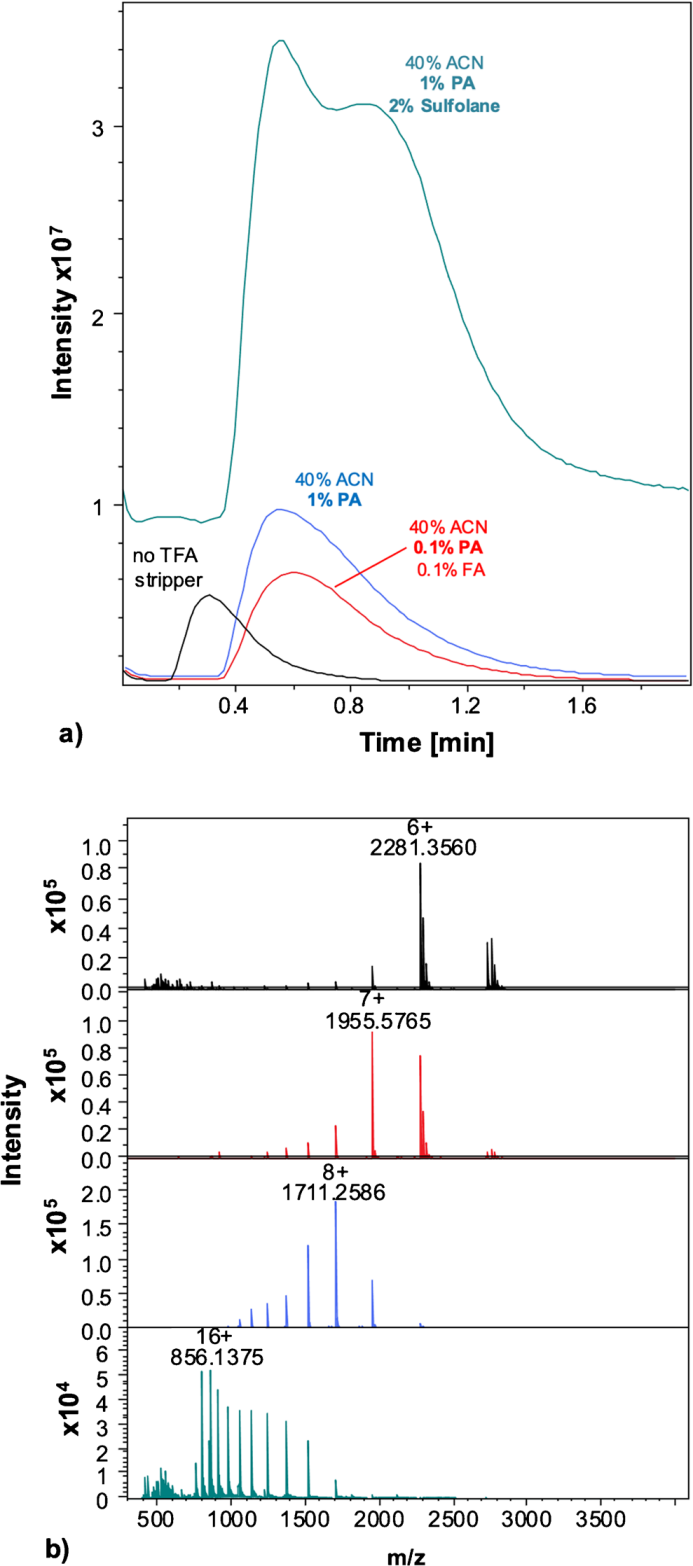
Flow injection ESI-MS of RnA (0.1 mg/mL) in ACN-water 40:60 (v/v) containing 0.1% TFA. **a** TIC obtained for RnA without μEMPIS (black) and with μEMPIS using a stripper solvent of ACN-water 40:60 (v/v) containing 0.1% FA and 0.1% PA (red), 1% PA (blue), and 1% PA and 2% sulfolane (green). **b** Mass spectra obtained for RnA using the stripper conditions mentioned under **a** (corresponding colors). Other conditions: injection volume, 2 μL; eluent and stripper flow rate, 0.2 mL/min. Further conditions, see Experimental section. Extracted ion currents (EIC) for different RnA charge states under the different conditions in Fig. 3a are reported in Fig. S1 of the ESM. See Fig. S2 of the ESM for a closer comparison of the MS spectra with 1% PA and 1% PA and 2% sulfolane

Different solvents and additives were used for the stripper flow and are described in the figures (Fig. 2 illustrates the effects of different stripper flow compositions; other experimental conditions tested are reported in section S3 of the ESM) of the respective experiments. The stripper flow rate was about 6 times the eluent flow rate.

### Mass spectrometry

**Fig. 3:**
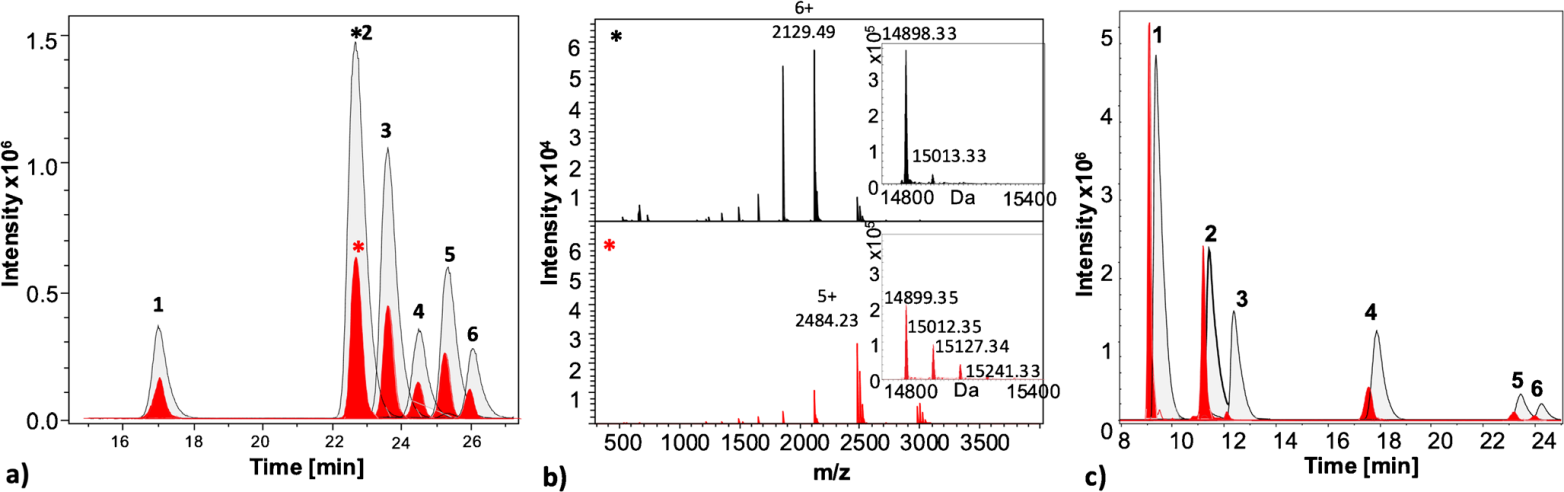
**a** Extracted ion current (EIC) of the ribonuclease glycoforms: RnA (1), RnB1 (2), RnB2 (3), RnB3 (4), RnB4 (5), RnB5 (6) obtained from a HILIC-MS run using of 0.4 μg of a mixture of RnA and B with (black) and without μEMPIS device (red). **b** Mass spectra of ribonuclease B1 (*) and inserts showing the corresponding deconvoluted mass spectra and the relative abundance of TFA adducts. The stripper flow composition was of 70% ACN with 0.1% FA and 1% PA. The runs with and without μEMPIS are time-aligned to facilitate the comparison (shifted about 0.5 min). A summary from the data from the extracted ion current for the single protein glycoforms is reported in Fig. S2 of the ESM. The EICs are obtained summing the intensities of the *m/z* for 7+, 6+, 5+, 4+ mass of each proteoform of RnA. RnA (1521.3, 1711.2; 1955.6; 2281.3 *m/z*), RnB1 (1656.5; 1863.4; 2129.5; 2484.2 *m/z*), RnB2 (1674.5; 1883.7; 2152.6; 2511.2 *m/z*), RnB3 (1692.5; 1903.9; 2175.8; 2538.2 *m/z*), RnB4 (1710.5; 1924.2; 2198.9; 2565.3 *m/z*), RnB5 (1728.5; 1944.4; 2222.1; 2592.3 *m/z*). All EICs are extracted with a mass range of ± 0.5 m/z. Other experimental conditions are reported in the experimental section. **c** EIC of the HILIC-MS separation of a protein mixture composed of (1) Ubi, (2) Cyt *C*, (3) Lys, (4) RnA, (5) RnB1, (6) RnB2 (each 0.2 mg/mL; 1 μL injected) with (black trace) and without (red trace) using the device. The TIC for the separation is reported in ESM Fig. S2; the chromatographic conditions are described in the Experimental section. The EICs are obtained summing the intensities of the *m/z* for the main form of the protein. Ubi (714.71; 779.50; 857.45; 952.50; 1071.56; 1224.35; 1428.41; 1713.90; 2142.12 *m/z*), Cyt C (727.95; 773.39; 824.88; 883.72; 951.63; 1030.93; 1124.47; 1236.81; 1374.13; 1545.77; 1766.44; 2060.68), Lys (1192.96; 1301.32; 1431.45; 1590.39; 1788.94; 2044.37; 2385.09), RnA (1521.33; 1711.25; 1955.57; 2281.33), RnB1 (1656.49; 1863.42; 2129.48; 2484.23), RnB2 (1674.49; 1883.67; 2152.63; 2511.24). All EICs are extracted with a mass range of ± 0.5 *m/z*. Other experimental conditions are reported in the Experimental section. Results of the area, height, signal to noise, full width at half height are reported in Table 1. The corresponding TIC are in Fig. S5 of the ESM

A Bruker Daltonics maXis HD high-resolution quadrupole time-of-flight (qTOF) mass spectrometer (Bremen, Germany) was used in positive-ion mode. Operating conditions are as follows: nebulizer: 0.8 bar; dry gas: 8 L/min; nitrogen dry temperature: 220 °C; quadrupole-ion and collision cell energies: 5 and 10 eV, respectively; collision cell RF: 2000 Vpp; in-source CID (isCID): 30 eV; funnel RF: 400 Vpp; multipole RF: 800 Vpp; transfer and prepulse storage times: 190.0 and 20.0 μs, respectively. The monitored mass range was 300–4000 *m/z*. Data analysis was done using Compass data analysis (4.3) from Bruker and deconvolution of protein mass spectra was done using the Maximum Entropy algorithm. All EICs are extracted with a mass range of ± 0.5 *m/z*. The LC-MS data (Fig. 3) have been deposited to the MassIVE Archive identifier MSV000086798 (ftp://massive.ucsd.edu/MSV000086798/).

## Results and discussion

### Design of the microfluidic ESI-enabling Mobile Phase Ion-Strip (μEMPIS)

We designed a multichannel microfluidic device (μEMPIS) to alleviate the signal suppression and adduct effects of TFA in HILIC of intact proteins operating at flow rates of 0.1 to 0.2 mL/min. A schematic representation of the device design and its operating principle are depicted in Fig. 1.

The μEMPIS design is based on an earlier described microfluidic ion suppressor for ion chromatography [21], modified to accommodate multiple parallel channels to increase its ion-exchange capacity. This ion stripper device features a bifurcating channel layout in which the effluent flow is equally distributed over eight microfluidic channels with dimensions of 60 mm × 200 μm × 300 μm (length × depth × width). The design of this distributor was previously optimized using computational fluid dynamics and experimental verification [23]. This design allows to generate a relatively large membrane contact area (approximately 50 mm^2^), while reducing the linear flow velocity and hence increase the analyte residence time (here approximately 0.15 min at 0.2 mL/min) with respect to a design featuring a single long and narrow meandering channel. On the other hand, the 8-channel design causes less band broadening than a single broad channel offering the same membrane contact area and residence time. Notably, the 8-channel design also provides essential support for the flexible membrane. When spanning a single wide channel of, e.g., 1 mm or more, such an unsupported flexible membrane could restrict the flow in the channels when pressure differences are introduced between the stripper flow side and the effluent side.

During its operation, TFA ions from the column effluent are exchanged for counterions, e.g., formate or propionate, provided by the stripper flow solution, which is applied in counterflow. TFA can diffuse through the membrane towards the stripper channel driven by the concentration gradient across the membrane, while the counterions migrate to the effluent channel to balance the charge (and to reduce its concentration gradient). The counter flow ensures that the concentration gradient of TFA between the channels is maximized near the outlet of the effluent channels and therefore favors the exchange of the residual TFA present in the effluent. The pH in the effluent remains low enough to keep the proteins positively charged, ensuring they are repulsed from the positively charged membrane surface. The presence of acetonitrile in the effluent minimizes hydrophobic interactions of the proteins with the membrane.

To asses potential protein losses due to adsorption on the membrane of the μEMPIS device, we performed flow injection analysis using mobile phase composition that mimic the chromatographic conditions commonly used in denaturing chromatography. In particular, we used mobile phases having low (10% ACN + 0.1% TFA) and high percentages of ACN (45% ACN + 0.1% TFA). Experiments were performed with and without device using reference proteins (BSA, trypsinogen and lysozyme) and UV detection (Table S1). The presence of high percentage of ACN (above 45%) was important to ensure sufficient recovery. Recoveries were between 90 and 100% when using 45% of ACN (Table S1). Therefore, we decided to limit the scope of the device to HILIC applications where the ACN % is maintained above 45%.

After having established conditions that prevent protein adsorption, we used flow injection ESI-MS experiments to evaluate the effect of the experimental conditions (stripper flow composition, flow rate) on the efficiency of the μEMPIS in increasing MS signal intensity and reducing adduct formation. This was done by injecting 2 uL of solution of RnA in ACN-water 40–60 (v/v) containing 0.1% TFA in an eluent of the same composition with a flow of 0.2 mL/min and different stripper flow compositions. The stripper solutions were based on 40% ACN with various additives: 0.1% propionic acid (PA) and 0.1% formic acid (FA), 1% PA, and 1% PA and 2% sulfolane. Both PA and sulfolane have been described as capable of reducing TFA signal suppression and adduct formation (propionic acid [7, 13, 19] and sulfolane [24]). The corresponding protein total ion chromatograms and mass spectra are reported in Fig. 2.

Under all conditions, the incorporation of the microfluidic stripper device increased the band width by about 1.27× (peak width at half height, FWHM, increased from 0.259 to 0.329 min) as can be visually observed comparing the black trace with the other traces in Fig. 2a. The increase of peak width results from the increased dispersion volume introduced by the μEMPIS device (32 μL). However, the increased peak width was accompanied by an improvement on the MS detection in terms of signal increase and removal of TFA adducts. No increase of peak asymmetry (e.g., tailing) was observed (in both cases, we observed a value of approximately 1.7).

When using a stripper solvent containing 1% propionic acid (PA), a 2× increase of the TIC signal intensity for RnA was obtained (Fig. 2a). Moreover, the corresponding mass spectrum of RnA showed a shift to higher charge states (maximum of the charge state distribution shifting from [M + 6H^+^]^6+^ to +[M + 8H^+^]^8+^) as well as the absence of protein-TFA adduct peaks. Results obtained with a lower concentration of PA (0.1%) showed a similar shift in protein charge state but had a reduced effect in terms of adduct removal. This suggests that a higher concentration gradient of PA is needed to favor the TFA exchange to compensate for the different pKa between the acids (0.23 for TFA vs 4.88 PA). Moreover, the introduction of PA in the ESI solvent is likely to reduce the ionization suppression and favor the removal of adducts during the ESI of the protein. The higher boiling point of PA with respect to TFA has been indicated as a possible reason for its favorable TFA adduct removal behavior [12, 18].

Additionally, we evaluated the use of a supercharging agent (sulfolane [25]) in the stripper flow to enhance the protein signal. Supercharging agents have shown potential in increasing MS signal when using TFA-based mobile phases. However, the direct addition of reagent like sulfolane directly in the mobile phases can create permanent changes to the column surface disrupting chromatographic performance [24].

A solution containing 2% (w/v; g/100 mL) sulfolane together with 1% propionic acid (PA) was mixed with the stripper flow, leading to a substantial increase (15×) of the intensity of the TIC signal for RnA. A significant shift of the protein charge state distribution (maximum signal at +16 when using sulfolane as compared to +8 for the stripper flow solution containing 1% PA only) was observed (Figs. S2 and S3 of the ESM). However, the gain in MS signal when using sulfolane was compromised by an increase of the background signal. This is likely to be a consequence of the formation of protein-sulfolane adducts in the gas phase [26].

Interestingly, the TIC peak profile of the RnA peak presented a bimodal distribution. To better understand this last effect, we extracted ion currents from different charge states (+17, +14, and + 10). The corresponding extracted ion currents showed a bimodal distribution profile only for the higher charge states (results reported in ESM Fig. S4). This seems to exclude unspecific adsorption of the protein to the suppressor membrane due to the sulfolane (this should happen for all the protein charge states in case of adsorption) but to be a consequence of a change in the supercharging efficiency depending on the resulting from a different ratio between protein and supercharging agent. We concluded that sulfolane can be used in combination with the μEMPIS device; however, including this generated additional protein-sulfolane adducts and we preferred continuing our experiments excluding this.

Next, the effect of the flow rates of the eluent and stripper solvent was evaluated. Lowering eluent flow rates from 0.2 to 0.1 mL/min increased the resident time of the analytes in the microfluidic device (by factor 2), allowing for a higher exchange rate and a corresponding signal increase (Fig. S4 of the ESM). Similarly, a higher stripper flow was beneficial (data not shown), helping to increase the concentration gradient between the two solvents. For the stripper solvent, a flow rate of about 6× the mobile phase flow rate was selected.

### Application of the μEMPIS to HILIC-MS intact protein separations

The conditions obtained from this optimization were used to apply the μEMPIS device to the separation of protein mixtures. Here, we tested HILIC separations based on water-ACN mobile phases containing 0.1% TFA. Figure 3a and b show the HILIC-MS analysis of RnA and RnB employing a TFA-containing eluent without (red trace) and with (black trace) the μEMPIS device. The separation was run at 0.1 mL/min using a gradient and a stripper flow of 0.6 mL/min with a composition of 70% ACN containing 0.1% FA and 1% PA (i.e., close to the HILIC separation conditions during elution).

In the HILIC separation, the different proteoforms of ribonuclease are resolved. The first eluting peak corresponds to the non-glycosylated protein (RnA) followed by its glycoforms (RnB). RnB is N-glycosylated with a high mannose glycan consisting of 5–9 mannose units (RnB1–5, respectively). In HILIC, the different glycoforms are separated according to the number of mannose units (low to high). Notably, the addition of TFA is essential for achieving the efficient separation of the glycoforms of RnB; i.e., no glycoform resolution is obtained when FA is used instead in the mobile phase [5].

The extracted ion currents (EICs) in Fig. 2a are obtained summing the intensity of the protein peak in 4 charge states from +4 to +7 (± 0.5 *m/z*), allowing to remove the signals from the protein-TFA adducts. Our results show a clear improvement in sensitivity when using the μEMPIS, with a peak area increase of about threefold and more than twofold in signal-to-noise ratio (S/N) (results reported in Table S2 of the ESM). Moreover, the obtained mass showed a shift of the protein ions to higher charge states when incorporating the TFA stripper (Fig. 3b). The TFA adducts account for about 50% of the deconvoluted protein signal (red spectra, insert in Fig. 3b) in the analysis without μEMPIS, whereas they account for less than 10% when the device is present (black spectra, insert in Fig. 3b). Notably, the introduction of the μEMPIS increases the peak widths (about 1.5×, e.g., from 0.280 to 0.415 min FWHM for RnA peak), following the trend observed in FIA.

The peak area and width increased when using the μEMPIS (Table 1; area from 3 and up to 32× and peak width between 1.5 and 3×), while the asymmetry of the peaks did not change. The signal gain (area and height) varied depending on the protein. Proteins like Ubi and Cyt C did not present significant TFA adducts without the TFA stripper; therefore, the gain in signal was limited. Instead for RnA and Lys 4 to 8, TFA adducts were observed (Figs. S6 and S7 of the ESM). Here, the TFA stripper helped in reducing the dispersion of the signal, significantly increasing the height and area of the extracted ion current of the protein (i.e., 32× area and 13× height increase for Lys). Moreover, proteins eluting closer to the start of the gradient (at higher % of ACN) were more severely affected by the band broadening from the microfluidic device. This seems to indicate the presence of unspecific interactions with the μEMPIS device that are somehow more relevant for less polar proteins (Ubi, Cyt C, eluting earlier in HILIC).

**Table 1:** Analysis of the increase of area, height, and full width at half height (FWHW) of the EIC the HILIC-MS analysis of proteins reported in Fig. 3. The results indicate the ratio of increase and are obtained by dividing the result (e.g., area) obtained with the μEMPIS device with the one obtained without the device

Peak	Area (with μEMPIS/without μEMPIS)	Height (with μEMPIS/without μEMPIS)	FWHM’ (with μEMPIS/without μEMPIS)
(1) Ubi	2.68	0.91	2.96
(2) Cyt C	2.18	0.98	2.23
(3) Lys	32.24	13.25	2.24
(4) RnA	4.07	2.72	1.41
(5) RnB1	5.23	3.31	1.47
(6) RnB2	5.34	3.36	1.51

## Conclusions

Here, we present proof of principle results demonstrating the feasibility of alleviating ion suppression by TFA-containing mobile phases in HILIC using a post-column multichannel microfluidic device (μEMPIS). The μEMPIS allowed to exchange TFA ions across a anion-exchange membrane, replacing them with formic and propionic acid ions and favoring ionization. The use of the μEMPIS device reduced the amount of protein-TFA adducts, but it did not eliminate the adducts for all the charge states. However, our results prove that signal improvement in terms of peak area and height can be achieved using this approach.

The capacity is one of the current limiting factors of the device. Fluid-dynamic investigations should be performed to maximize the exchange surface while minimizing dispersion, e.g., by making use of dual membranes, or hollow fibers. Moreover, membranes based on chemistries with reduced lipophilicity should be studied to reduce potential interactions with the membrane that lead to unwanted increase of peak widths and extend the application to RPLC-MS.

## Supplementary information

ESM 1 (PDF 394 kb)
