# Supplementary material for: Microfluidic ion stripper for removal of trifluoroacetic acid from mobile phases used in HILIC-MS of intact proteins
Source: Anal Bioanal Chem. 2021 May 28;413(17):4379–86. doi: 10.1007/s00216-021-03414-4 (PMC8245364; doi:10.1007/s00216-021-03414-4)
Supplement: Supplementary file 1 — (PDF 394 kb) [file 216_2021_3414_MOESM1_ESM.pdf]

Supplementary Information

Microfluidic ion stripper for removal of trifluoroacetic acid from mobile phases used in HILIC-MS of intact proteins

Sam Wouters [a], Sebastiaan Eeltink [a*], Rob Haselberg [b,c], Govert W. Somsen [b,c], Andrea F.G. Gargano [b,c*]

[a] Vrije Universiteit Brussel (VUB), Department of Chemical Engineering, B-1050 Brussels, Belgium
[b] Center for Analytical Sciences Amsterdam, Science Park 904, 1098 XH Amsterdam, The Netherlands
[c] Division of BioAnalytical Chemistry, Amsterdam Institute of Molecular and Life Sciences, Vrije Universiteit Amsterdam, de Boelelaan 1085, 1081HV Amsterdam, The Netherlands

KEYWORDS: Microfluidics, Ion suppression, Ion-pairing, LC-MS, intact protein analysis

*a.gargano@uva.nl, seeltink@vub.ac.be
Dr. Andrea Gargano
https://orcid.org/0000-0003-3361-7341
Analytical Chemistry
Center for Analytical Chemistry Amsterdam
Universiteit van Amsterdam
Tel: +31-20-525 6531
Science Park 904 |1098 XH Amsterdam
Building C2, room 258

Table of Contents

S1 Effect of TFA in LC-MS of intact proteins 3

S2 Investigation of protein adsorption on µEMPIS device 5

S3 Influence of regenerant composition and flow rate on µEMPIS performance 7

S4 Analysis of sensitivity gain by µEMPIS in HILIC-MS................................... 10

S5 Details of the mass spectrum in Figure 3c and Figure S.4 12

S1 *Effect of TFA in LC-MS of intact proteins*

HILIC has shown to be useful for the separation of intact proteins, providing orthogonal selectivity with respect to RPLC. For both techniques, the addition of TFA to the mobile phase significantly improves intact protein peak shape, yielding improved resolution. TFA is an effective ion-pairing agent, circumventing adverse secondary electrostatic interactions of proteins with the stationary phases in both RPLC and HILIC. However, when using ESI-MS TFA can leadleads to significant reduction of the protein MS signals often accompanied by protein-TFA adducts.

Exchanging TFA with more MS-compatible eluent additives, such as FA, enhances the MS response but also significantly reduces the separation performance. This is demonstrated in Figure S.1, which shows chromatograms and mass spectra obtained for a protein mixture comprising RnA and B, Cyt *C*, Ubi, Lys, Trans, and CA when analyzed by RPLC and HILIC-MS applying FA and TFA as mobile-phase additives, respectively. TFA-containing mobile phases yield significantly narrower and also more symmetric peaks when the intact proteins are analyzed in HILIC and RPLC (Figures S.1a and S.1b) resulting in higher peak capacities (76 *vs.* 32 for RPLC using TFA and FA, respectively). Moreover, TFA influences protein retention compared to when FA is present in the mobile phase. TFA-protein ion-pairing overall increases analyte lipophilicity, enhancing RPLC retention (based on hydrophobic interaction) and decreasing HILIC retention (based on polar interactions). Additionally, TFA causes a significant loss of protein signal intensity, as is clear from the substantially decreased peak heights and areas obtained for the proteins when using a mobile phase with TFA (see S.1a/b). For example, when changing from TFA to FA in RPLC analysis, the peak area of Cyt *C* increases by a factor 10 from about $2 \cdot 10^7 to\ 2 \cdot 10^8$counts·min. Similarly the absolute signal for RnA decreases by one order of magnitude (S.1c/d; the MS spectrum intensity of RnA). Moreover, in the presence of TFA TFA-protein adducts/clusters are observed (mass differences of 114), together with lower charge states (i.e. higher m/z).

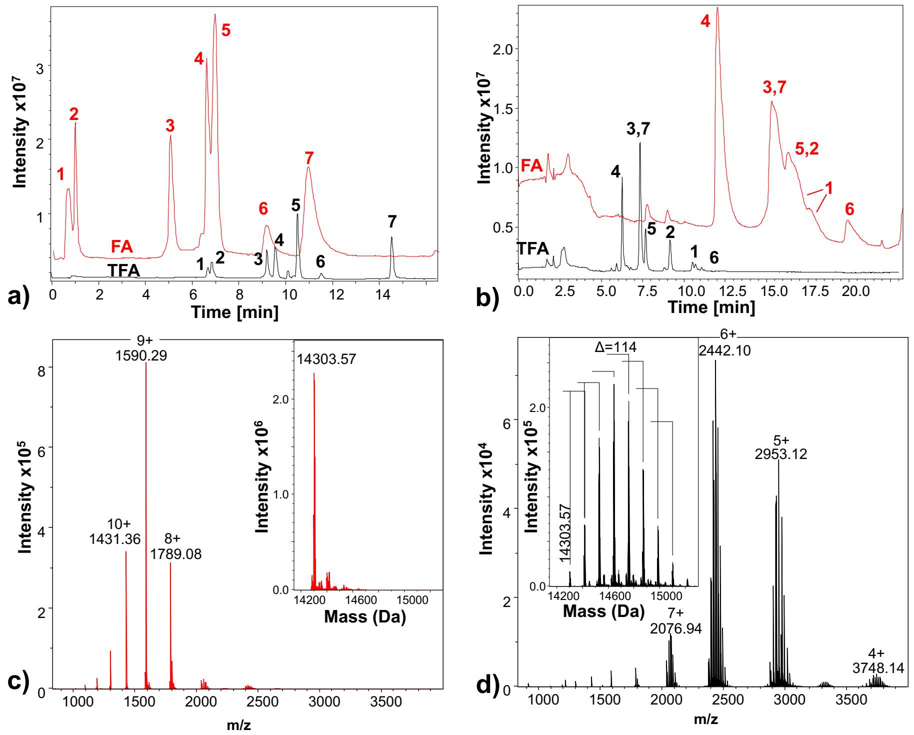

Figure S1: Total-ion chromatograms (TICs) obtained during RPLC-MS (a) and HILIC-MS (b) analysis of a protein mixture using a mobile phase with 0.1% TFA (black) or 0.1% FA (red). Peaks, (1) RnA, (2) RnB, (3) Cyt *C*, (4) Ubi, (5) Lys, (6) Trans, (7) CA (each 0.2 mg/mL; 1 μL injected). Mass spectra for Lys obtained during RPLC-MS using a mobile phase with 0.1% FA (c) and 0.1% TFA (d). Inserts show the corresponding deconvoluted mass spectra (+114 in mass correspond to protein-TFA adducts).

S2 Investigation of protein adsorption on µEMPIS device

Material and methods

We set up these experiments to probe analyte-membrane interactions which could potentially result in retention (peak broadening), or carry-over. Diffusion of analytes into or through the membrane, or irreversible adsorption (membrane fouling), would also be sources of incomplete recovery.

For this purpose, we used a LC-UV setup (U3000 RSLC Cap, Thermo Fisher Scientific) having an autosampler and a stand-alone VICI valve with fixed injection volume (20 nL) and a UV detector (20 nL z-cell) operated at 210 nm at 20 Hz. In our test we injected bovine serum albumin, lysozyme from egg white, and trypsinogen from bovine pancreas (all from Sigma Aldrich, 5 mg/mL solutions in 25% ACN with 0.1% TFA) with and without the µEMPIS in the flow path (installed between the injector and detector). The respective peak areas were recorded in triplicate for two different solvent compositions (provided by the quaternary pump, isocratic), to serve as the reference values.

The µEMPIS device used had a single channel of dimensions 0.2x0.15x40 mm. The eluent channel was operated at 20 µL/min using different mobile phases (see table S1). Before the experiment it was conditioned applying ACN containing 0.1% FA as the regenerant flow at a flow rate of 100 µL/min.

Discussion

We observed that the percentage of organic content at which the analytes pass through the stripper influences the analyte recovery. This suggest that at low % of ACN the suppression of hydrophobic interactions is incomplete (see table S.1). We could obtain almost complete recovery when using more than 40% ACN (estimated as peak area difference between the experiments with and without µEMPIS). Similar results were observed with 80% ACN in the mobile phase (data not shown). This suggest that the device is likely to perform better under HILIC conditions respect to RPLC.

Table S1: Estimation of recovery of different test proteins (bovine serum albumin, trypsinogen, lysozyme) using the µEMPIS device at different regenerant and eluent compositions.

		**Mobile phase solvent composition of stripper flow and eluent**					
		45% ACN in water + 0.1 % TFA			10% ACN in water + 0.1 % TFA		
		peak area*		recovery	peak area*		recovery
	pI**	without device	with device		without device	with device	
BSA	5.6	50	45	91%	56	30.3	54%
TRYP	8.2	68	64	94%	75	61.7	82%
LYS	9.3	63	65	103%	70	63.3	91%

* mAU x min
** Theoretical

S3 Influence of regenerant composition and flow rate on µEMPIS performance

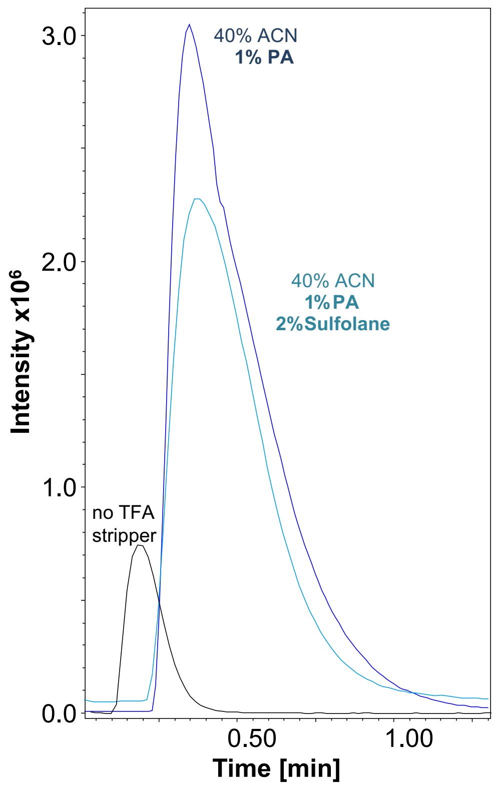

Figure S2: EIC of the ribonuclease A charge states, obtained under the conditions reported in Figure 3. The EICs are obtained summing the intensities of the m/z corresponding to charge states between +18 and +8 (805.83; 856.14; 913.14; 978.30; 1053.47; 1141.78; 1244.83; 1369.21; 1521.23; 1711.26; 1955.58; 2281.34) All EICs are extracted with a mass range of +/- 0.5 m/z. Other experimental conditions are reported in the experimental section.

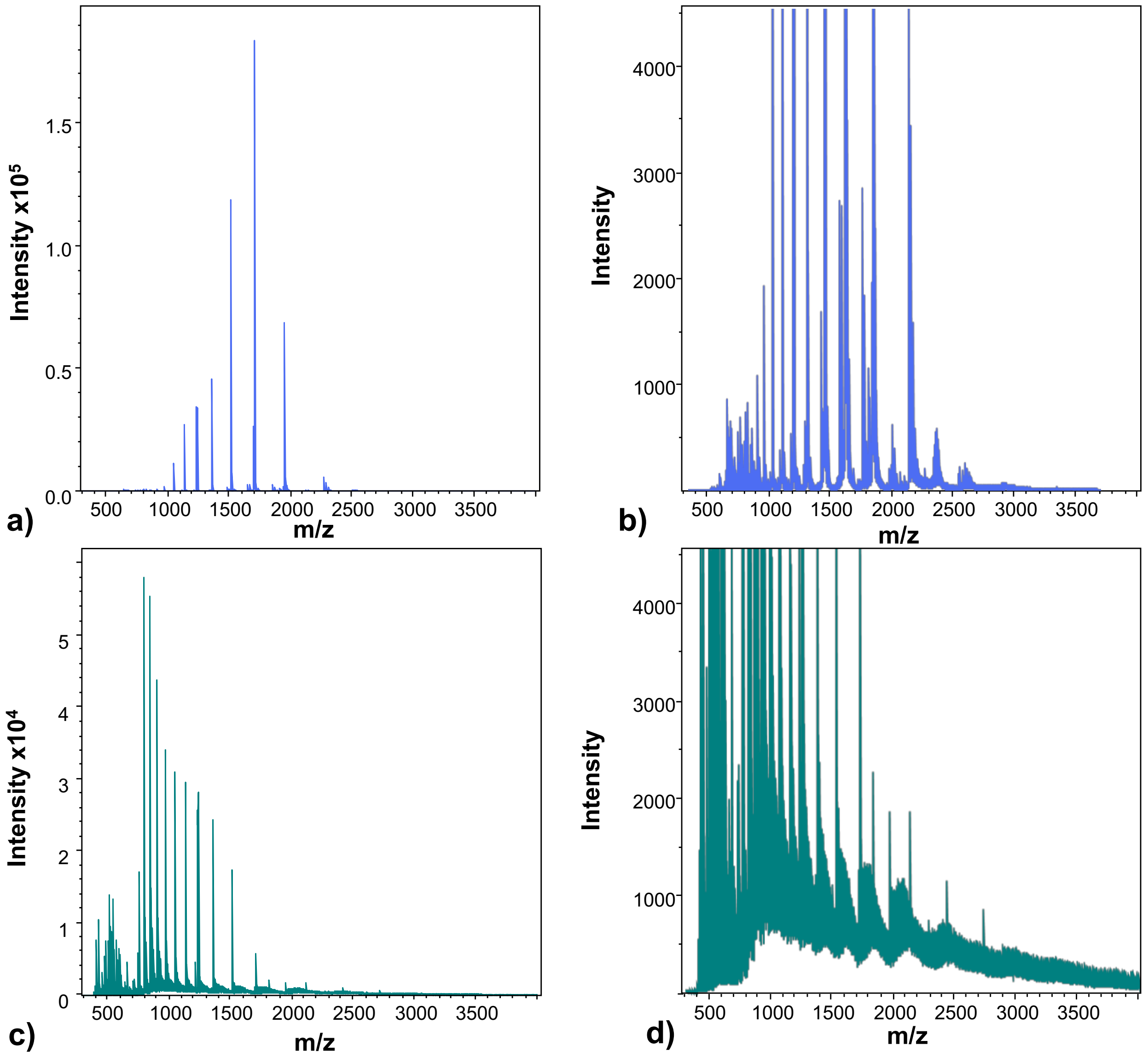

Figure S3: Detail of the MS spectrum of the ribonuclease A reported in Figure 2 using 40% ACN and 1% PA and 40% ACN 1% PA and 2% sulfolane.

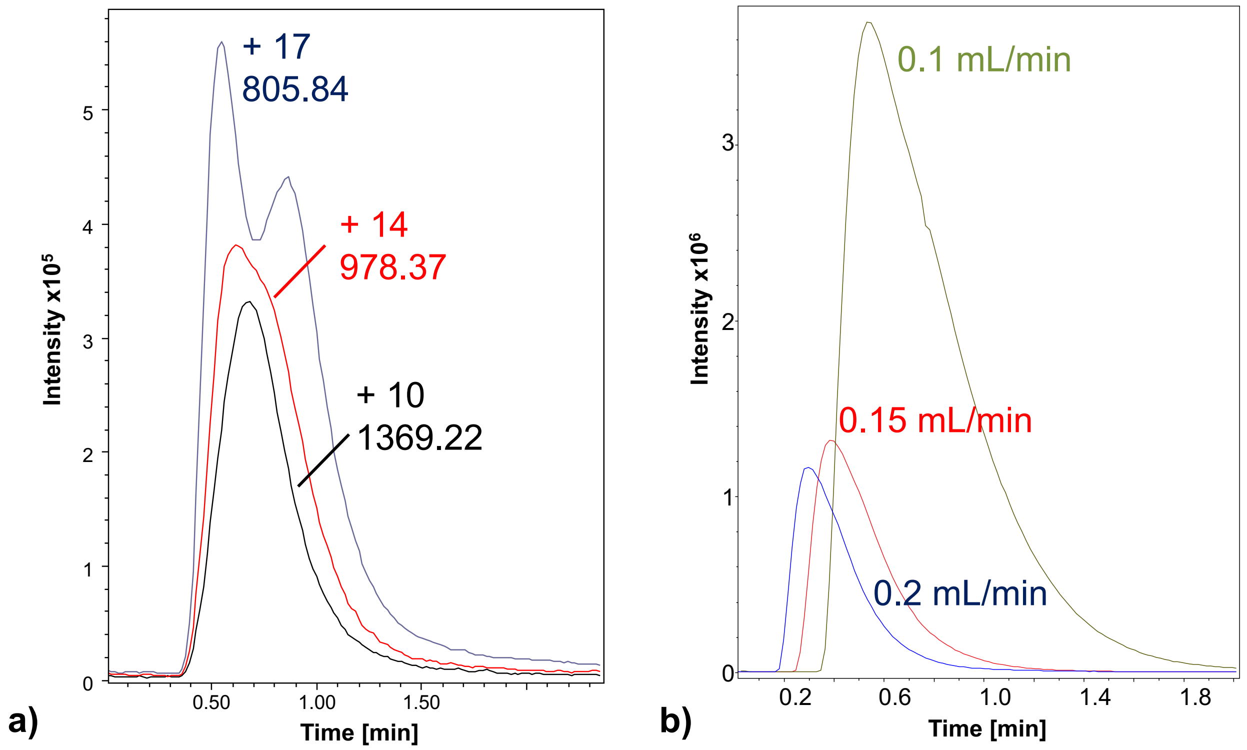

Figure S4: a) Extracted ion chromatogram (EIC) of for different charge states (+17, +14 and +10) of ribonuclease A (0.1 mg/ mL in 40% ACN 0.1% TFA, 2 µL injected) analyzed using a regenerant solution of 40% ACN 1% PA and 2% sulfolane (data from Figure 2of the manuscript). b) effect of the eluent mobile phase flow rate on the EIC response, using 40% ACN 1% PA as regenerant solution. Other conditions are the same as described in Figure S.2. The EICs are obtained summing the intensities of the m/z corresponding to charge states between +18 and +8 (805.83; 856.14; 913.14; 978.30; 1053.47; 1141.78; 1244.83; 1369.21; 1521.23; 1711.26; 1955.58; 2281.34) All EICs are extracted with a mass range of +/- 0.5 m/z. Other experimental conditions are reported in the experimental section

S4 Analysis of sensitivity gain by µEMPIS in HILIC-MS

Table S2: Results from the integration of the EIC of the single glycoforms of ribonuclease B analysed by HILIC-MS using TFA without (A), with µEMPIS (B) and % increased achieved using the µEMPIS

A)

No µEMPIS					
#	RT [min]	Area	I	S/N	FWHM [min]
1	17.706	3185194	167309	763	0.28
2	23.005	14528194	670060	2922.9	0.341
3	23.845	9477765	461397	2133.1	0.321
4	24.666	2859865	148183	658.1	0.298
5	25.394	4988195	268335	1178.5	0.289
6	26.05	2074275	120877	537.2	0.265

B)

With µEMPIS					
#	RT [min]	Area	I	S/N	FWHM [min]
1	17.59	10781709	4E+05	1298.7	0.415
2	22.95	51254404	2E+06	5940.5	0.506
3	23.83	34174864	1E+06	4326.8	0.463
4	24.67	8666163	4E+05	1212	0.392
5	25.46	18515220	6E+05	2226.5	0.444
6	26.13	7904116	3E+05	1111.1	0.403

C)

Increase by µEMPIS				
#	Area %	Intensity %	S/N %	FWHM %
1	238%	127%	70%	48%
2	253%	132%	103%	48%
3	261%	142%	103%	44%
4	203%	150%	84%	32%
5	271%	133%	89%	54%
6	281%	136%	107%	52%
Average	251%	137%	93%	46%

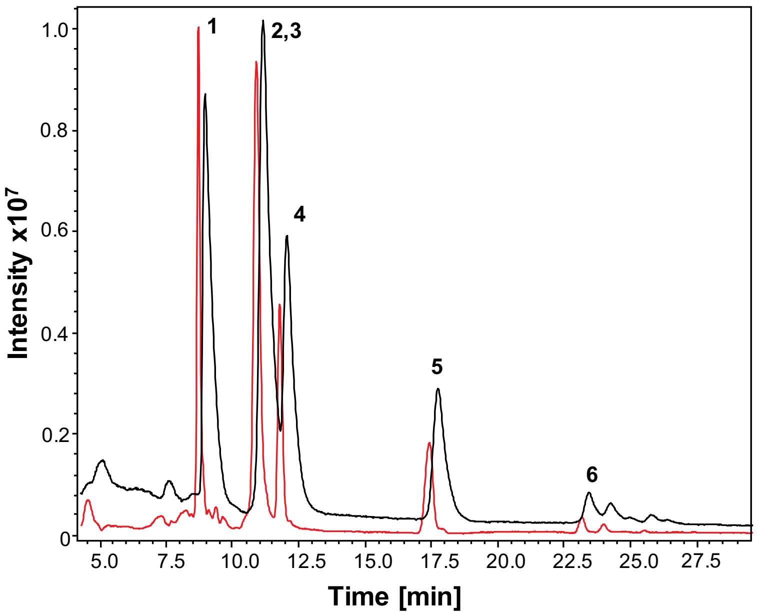

Figure S5: Total Ion Chromatogram (TIC) of the HILIC-MS separation of a protein mixture composed of: (1) Ubi, (2) Cyt C, (3) Lys, (4) CA, (5) RnA, (6) RnB with μEMPIS (black trace) and without (red trace). The corresponding EIC for the single proteins as well area and peakwidth are reported in Figure 2c and Table 1.

S5 Details of the mass spectrum in Figure 3c and Figure S.5

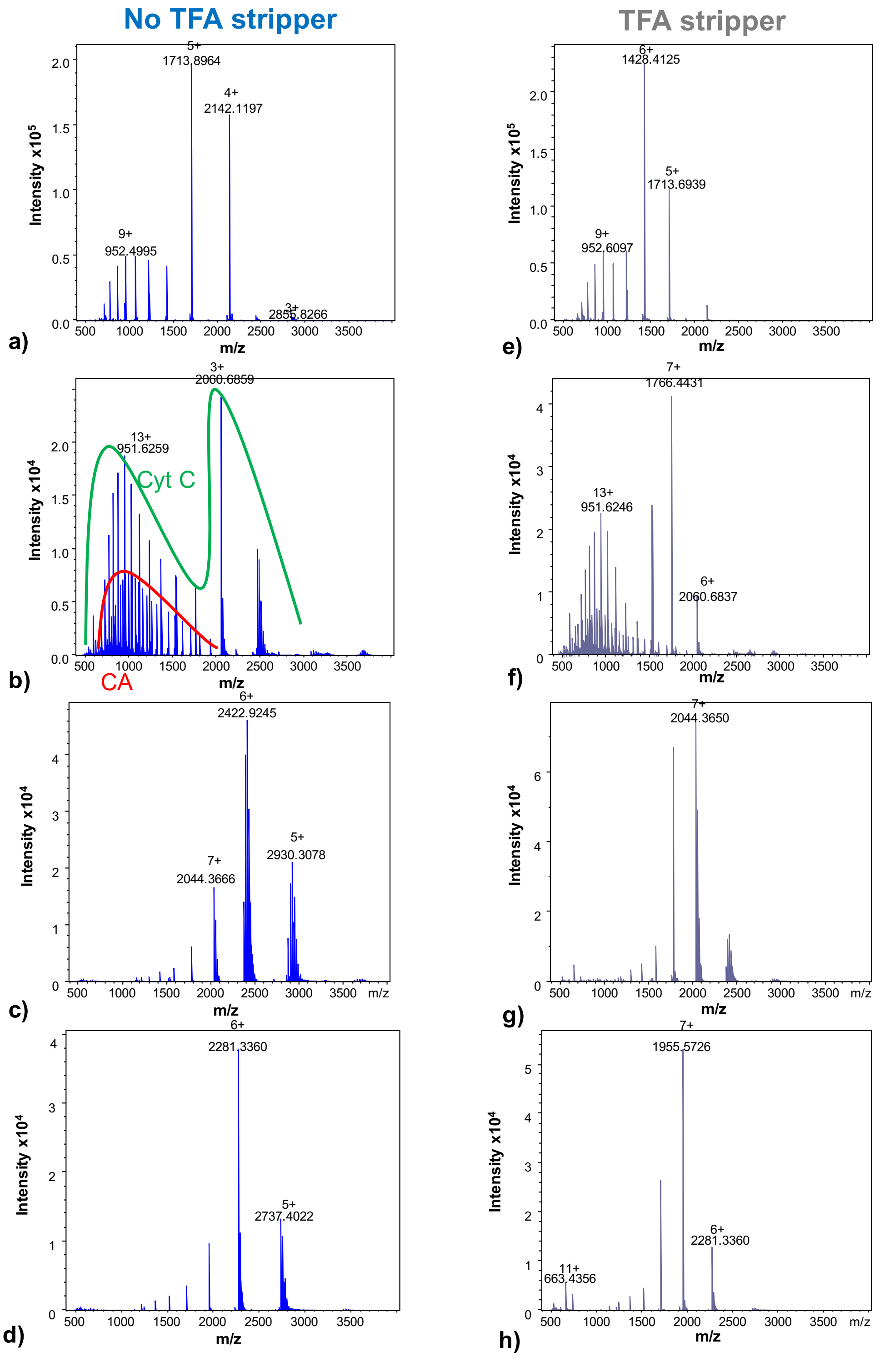

Figure S6: MS spectrum from the protein peaks for the HILIC-MS separation reported in Figure 4c. a) Ubi, b) Cyt C and CA, c) Lys, d) RnA with (gray) and without (blue)TFA stripper

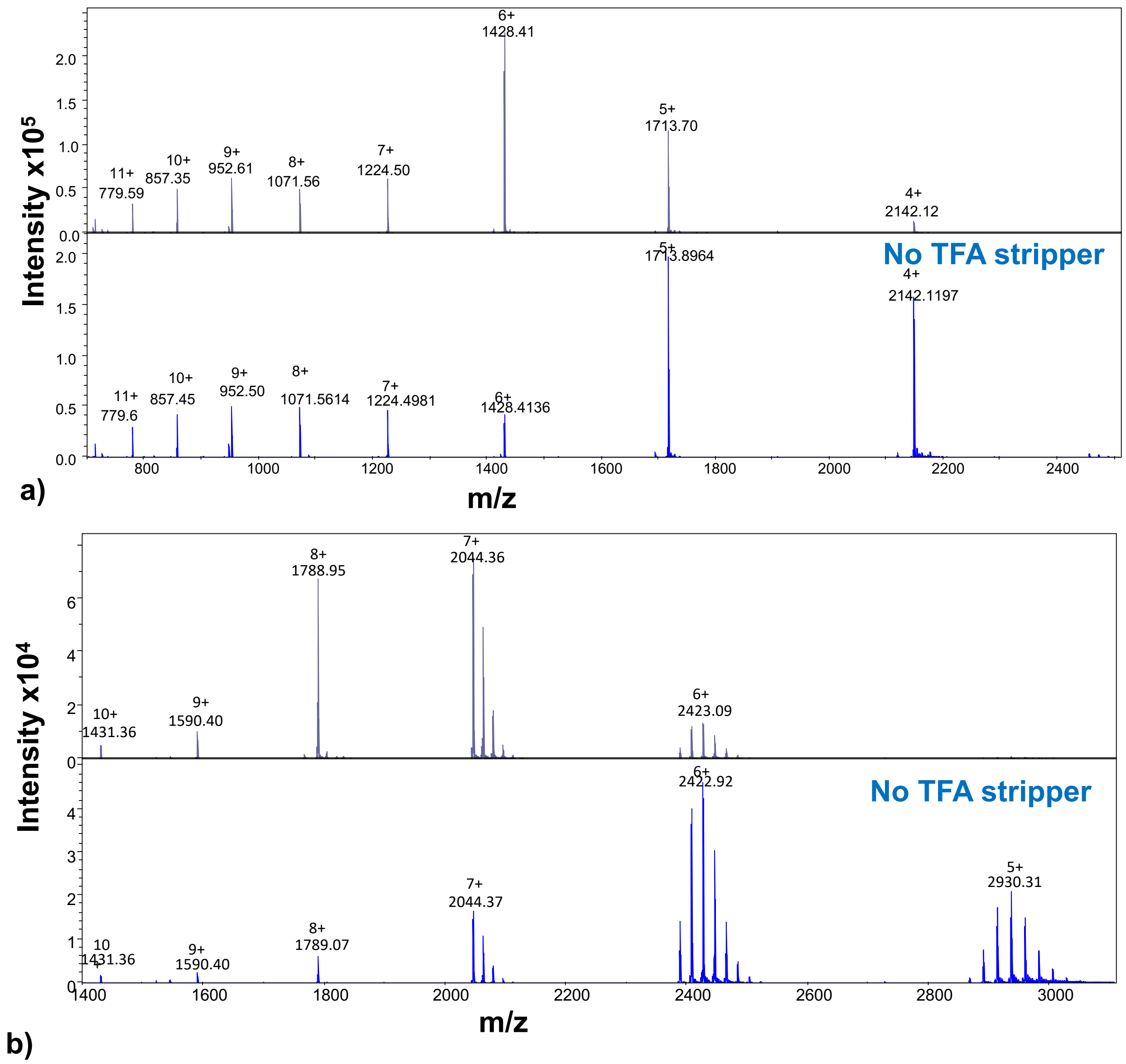

Figure S7: Detail of MS spectrum of a) Ubi and b) Lys reported in Figure S.6.
